# The role of cGAS-STING signaling in the development and therapy of head and neck squamous cell carcinoma

**DOI:** 10.3389/fimmu.2024.1451305

**Published:** 2024-09-04

**Authors:** Chengze Shao, Jiawen Chen, Bi Qiang, Junmei Ye, Fangrong Yan, Yongbo Zhu

**Affiliations:** ^1^ Department of Otolaryngology Head and Neck Surgery, The First Affiliated Hospital of Yangtze University, Jingzhou, Hubei, China; ^2^ Department of Oncology, The First Affiliated Hospital of Yangtze University, Jingzhou, Hubei, China; ^3^ Research Center of Biostatistics and Computational Pharmacy, China Pharmaceutical University, Nanjing, China; ^4^ School of Life Science and Technology, China Pharmaceutical University, Nanjing, China

**Keywords:** head and neck squamous cell carcinoma (HNSCC), cGAS-STING signaling pathway, cancer immunotherapy, viral infections, human papillomavirus (HPV)

## Abstract

The cGAS-STING signaling pathway plays a critical role in innate immunity and defense against viral infections by orchestrating intracellular and adaptive immune responses to DNA. In the context of head and neck squamous cell carcinoma (HNSCC), this pathway has garnered significant attention due to its potential relevance in disease development and progression. HNSCC is strongly associated with risk factors such as smoking, heavy alcohol consumption, and human papillomavirus (HPV) infection. The presence or absence of HPV in HNSCC patients has been shown to have a profound impact on patient survival and prognosis, possibly due to the distinct biological characteristics of HPV-associated tumors. This review aims to provide a comprehensive overview of the current therapeutic approaches and challenges in HNSCC management, as well as the involvement of cGAS-STING signaling and its potential in the therapy of HNSCC. In addition, by advancing the present understanding of the mechanisms underlying this pathway, Activation of cGAS–STING-dependent inflammatory signaling downstream of chromosomal instability can exert both anti-tumoral and pro-tumoral effects in a cell-intrinsic manner, suggesting individualized therapy is of great importance. However, further exploration of the cGAS-STING signaling pathway is imperative for the effective management of HNSCC.

## Graphical Abstract

HNSCC is primarily caused by smoking, heavy alcohol consumption, and HPV infection. Upon activation, the cyclic cGAS-STING signaling pathway plays a crucial role in HNSCC progression. cGAS recognizes cytosolic DNA, leading to the production of cGAMP. cGAMP then binds to and activates the STING receptor, triggering downstream signaling events. This activation results in the production of pro-inflammatory cytokines and type I interferons, promoting tumor growth and immune evasion. Targeting cGAS-STING signaling pathway is promising for HNSCC treatment. Abbreviations: HNSCC, Head and Neck Squamous Cell Carcinoma; HPV, Human Papillomavirus; cGAS, cyclic GMP-AMP synthase; STING, Stimulator of Interferon Genes; cGAMP, cyclic GMP-AMP.

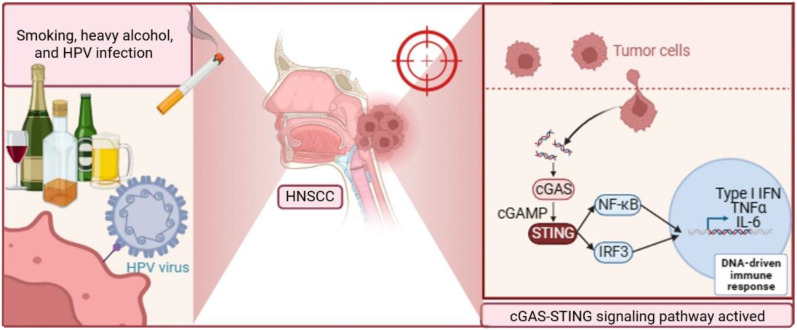

## 1 Introduction to HNSCC

Head and neck tumors comprise a variety of malignancies that occur in the neck, ear, nose, throat, and oral and maxillofacial regions. Most of these tumors arise from the mucosal epithelial cells of the oral cavity, pharynx, and larynx. More than 90% of head and neck tumors are squamous cell carcinomas, which are malignant tumors that arise from the epidermis or skin appendages (1). The incidence of Head and Neck Squamous Cell Carcinoma (HNSCC) has been steadily increasing over the years, with an estimated 1.08 million new cases predicted to be added annually, according to the Global Cancer Observatory (GLOBOCAN) (2, 3). In 2021, HNSCC is expected to account for 78% of deaths from head and neck tumors, and the incidence and mortality rates continue to rise (1). The majority of patients are diagnosed at a locally advanced stage, and the prognosis is generally poor, with approximately 50%-60% of patients experiencing local recurrence and 20%-30% developing distant metastases within two years. The five-year survival rate is typically less than 50% (4). HNSCC is a significant health problem due to its high incidence, high mortality rate, and the fact that it is the most common type of systemic tumor originating in the head and neck. HNSCC is more prevalent in men than in women, and men have two to four times the risk of developing HNSCC compared to women. The age of onset is generally over 50 years (5).

HNSCC is closely associated with smoking and heavy alcohol consumption, and countries with high tobacco and alcohol consumption have higher incidence rates (6). Human papillomavirus (HPV) is also a significant factor in the development of HNSCC (7). The HPV virus family consists of a group of DNA viruses, categorized into high-risk and low-risk types. The high-risk HPV viruses primarily include subtypes HPV16, which are the most common HPV subtypes associated with HNSCC (8).

In clinical practice, HNSCC is primarily classified into HPV-positive and HPV-negative cases. Oral and pharyngeal cancers are mainly associated with smoking and alcohol consumption, while HPV infection primarily causes oropharyngeal cancer. HPV infects the mucosal cells of the oral cavity and pharynx, integrating its DNA into the host cell genome, leading to abnormal cell proliferation and transformation, overexpression of oncogenes, and inactivation of tumor suppressor genes, ultimately resulting in malignant transformation and the development of HNSCC (9). Compared to HPV-negative HNSCC, patients with HPV-positive HNSCC exhibit distinct clinical features and treatment responses. HPV-positive patients are typically younger, have a more benign disease course, and tend to have a better prognosis. Additionally, HPV-positive HNSCC often demonstrates higher histological differentiation and fewer lymph node metastases. In clinical practice, HPV testing has become an important factor in evaluating the prognosis and selecting treatment strategies for HNSCC patients. HPV-positive HNSCC patients generally exhibit better responses to radiation therapy and chemotherapy, caused by limited DNA repair capacity (10), potentially requiring lower treatment doses and fewer treatment cycles (11). These findings underscore the urgent need for enhanced personalized treatment approaches for HNSCC.

## 2 Molecular mechanisms associated with cGAS-STING of HNSCC

### 2.1 cGAS-STING pathway overview

**Figure 1:**
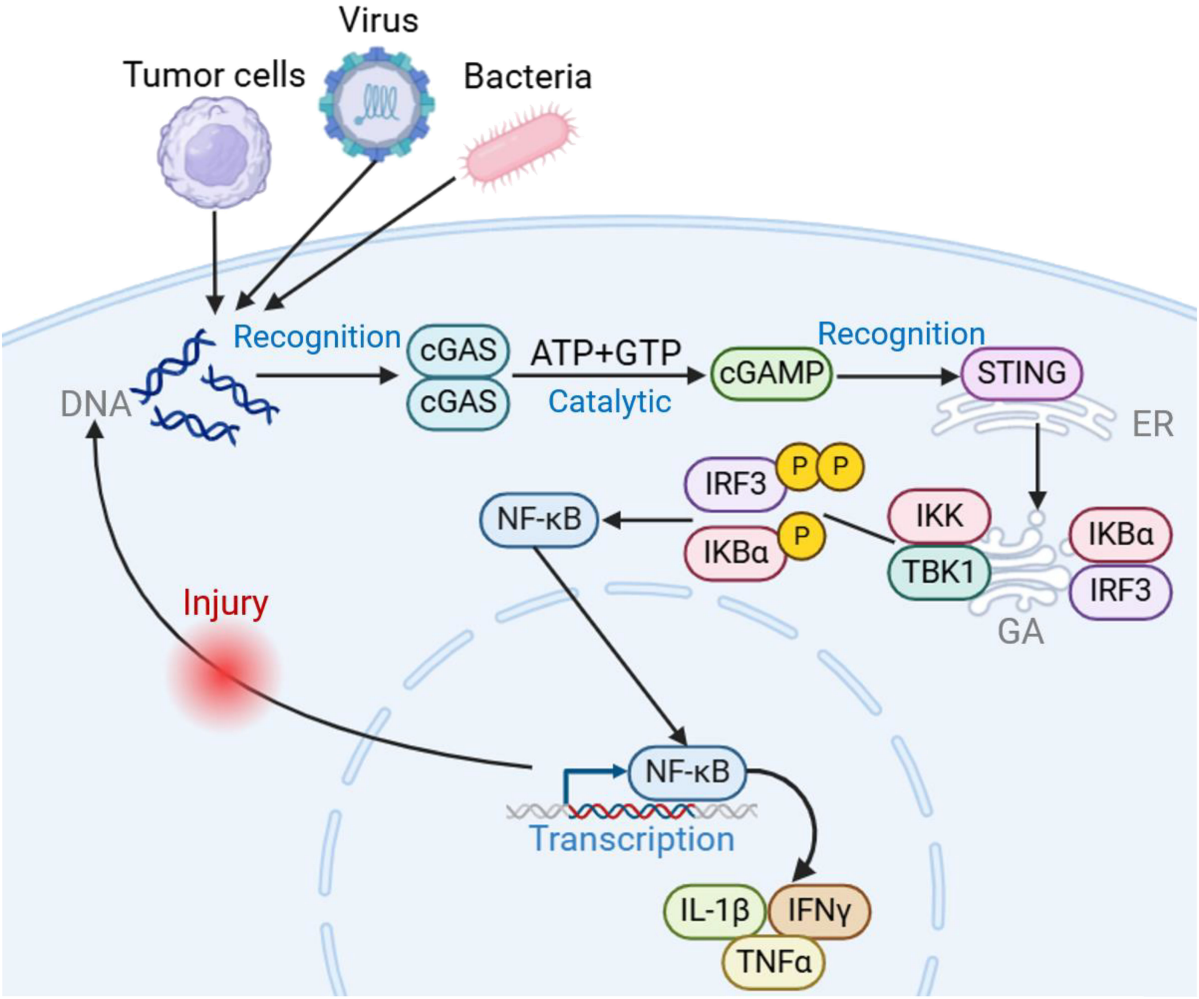
Introduction to cGAS-STING signaling pathway. Upon invasion by pathogens such as tumors, bacteria, and viruses, cGAS acts as a cytosolic DNA receptor, recognizing invading DNA and catalyzing the generation of 2’3’-cGAMP from ATP/GTP. STING acts as a downstream bridging molecule, recognizing cGAMP on the endoplasmic reticulum, dimerizing for transport to the Golgi apparatus, recruiting TBK1 and IKK, and phosphorylating IRF3, inducing an IRF3-dependent type I interferon response. At the same time, NF-κB is activated, releasing downstream pro-inflammatory cytokines involved in the adaptive immune response. Created using BioRender (https://biorender.com/).

The cGAS-STING signaling pathway serves as a critical component of the innate immune response, particularly in recognizing and responding to cytosolic DNA, including those derived from pathogens or cellular damage. Upon detection of cytosolic DNA, the cyclic GMP-AMP synthase (cGAS) catalyzes the synthesis of cyclic GMP-AMP (cGAMP), which then binds to and activates the Stimulator of Interferon Genes (STING) protein. STING, a transmembrane protein located primarily on the endoplasmic reticulum, undergoes a conformational change upon cGAMP binding, leading to its dimerization and subsequent activation. Activated STING then translocates from the endoplasmic reticulum to perinuclear vesicles, where it recruits and activates TANK-binding kinase 1 (TBK1) (12–14). TBK1 phosphorylates STING, leading to the activation of downstream signaling cascades, including the phosphorylation of interferon regulatory factor 3 (IRF3). Phosphorylated IRF3 translocates to the nucleus, where it induces the expression of type I interferons (IFNs) and other interferon-stimulated genes (ISGs), ultimately promoting an antiviral immune response (15). Additionally, STING activation also triggers the NF-kB signaling pathway. Upon activation, TBK1 not only phosphorylates IRF3 but also contributes to the activation of Ift kinase (IKK), which subsequently phosphorylates Ihos leading to its degradation. This degradation releases NF-kB, allowing it to translocate into the nucleus, where it induces the expression of pro-inflammatory cytokines and other immune-regulatory genes (**Figure 1**).

### 2.2 Impact of TP53/Notch and c-MET/HGF/EGFR pathways on cGAS-STING in HNSCC

**Figure 2:**
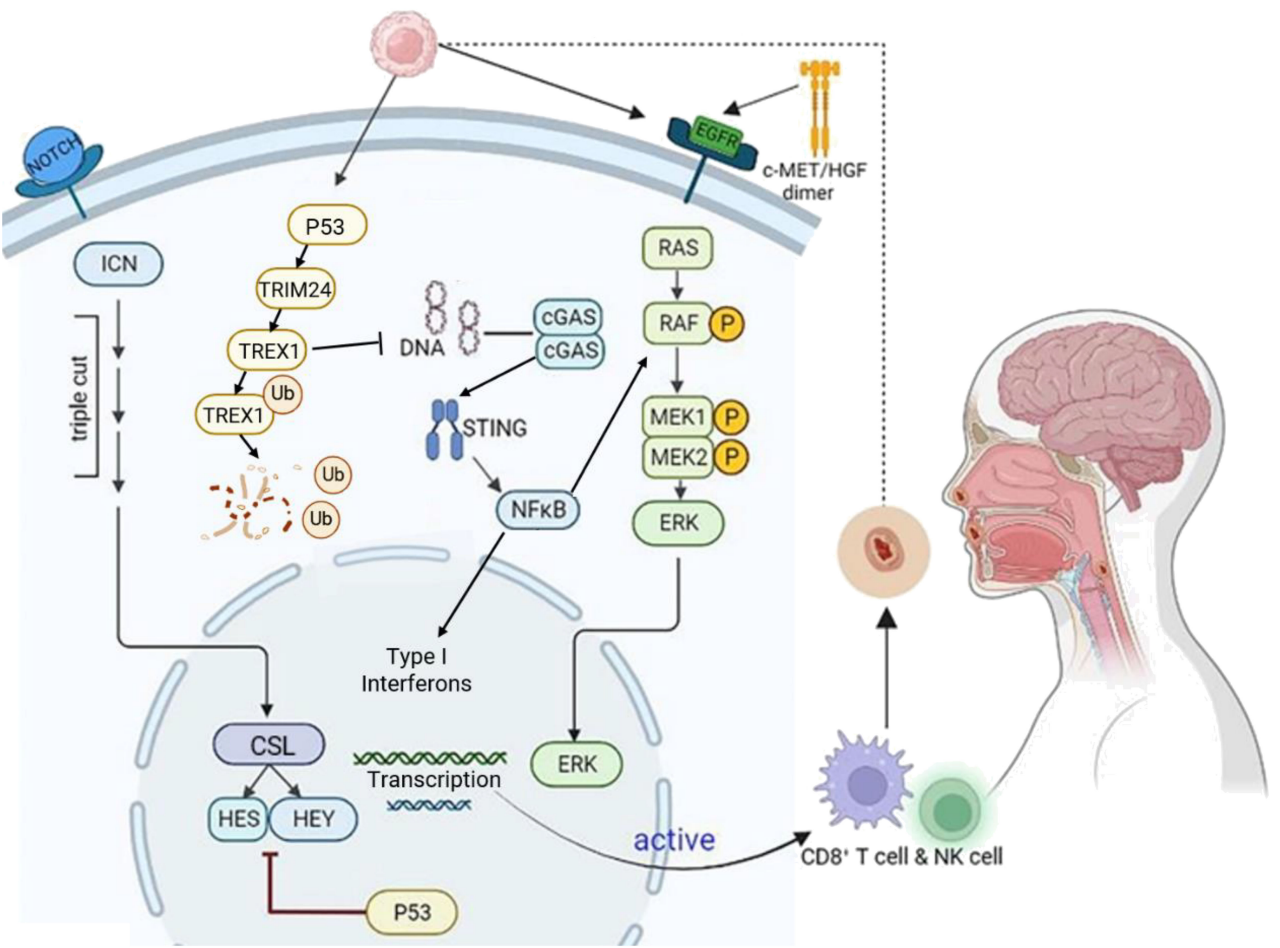
Molecular mechanisms in HNSCC development. HNSCC is characterized by the accumulation of long-term, multi-stage epigenetic alterations in multiple genes. Key mechanisms include TP53/Notch, c-Met/HGF/EGFR, and cGAS-STING signaling. TP53 mutations activate Notch signaling, leading to NICD/CSL complex formation and activation of HES, HEY, and other target genes. The c-Met receptor binds HGF, triggering EGFR pathway activation and promoting HNSCC invasion, migration, and angiogenesis. In the cGAS-STING pathway, cGAS recognizes HNSCC tumor DNA and generates cGAMP, which activates STING. This leads to NF-κB pro-inflammatory responses and IRF3-dependent type I interferon responses. Additionally, after p53 phosphorylates TREX1, TRIM24 ubiquitinates (Ub) TREX1, leading to its degradation and subsequent activation of the cGAS-STING pathway. Created using BioRender (https://biorender.com/).

Similar to most solid tumors, the development of HNSCC is a long-term, multistage process involving the accumulation of epigenetic changes, including mutations in multiple oncogenes and tumor suppressor genes. Several classical pathogenic mechanisms have been identified among the key genes associated with its pathogenesis, including the TP53/Notch signaling pathway, the c-MET/HGF signaling pathway (**Figure 2**).

P53, one of the first tumor suppressor proteins to be identified, plays a critical role in regulating cell growth, DNA repair, and induction of apoptosis. Poeta et al. (16) analyzed Tumor Protein 53 (TP53) gene mutations in the DNA of 560 HNSCC patients using gene microarray and high-performance liquid chromatography and found that TP53 mutations were present in 53.3% of HNSCC patients. The presence of TP53 mutations in HNSCC has been associated with poorer prognosis, including reduced survival rates and increased risk of disease recurrence, especially in the context of surgical and radiotherapy treatments (17). Cell line study has shown that p53 induces the degradation of TREX1 through the ubiquitin ligase TRIM24. The degradation of TREX1 leads to the accumulation of cytoplasmic DNA, which activates the cGAS-STING pathway and results in the induction of type I interferons. This means that p53 utilizes the cGAS-STING innate immune pathway to exert both intrinsic and extrinsic tumor suppressive activities (18).

The *Notch* gene encodes a highly conserved class of cell surface receptors, mutations in the *Notch* gene can either promote or inhibit tumor growth, depending on the location and cell type of the tumor. In head and neck tumors (19), *Notch1* inactivating mutations occur in 10%-15% of cases, making it the second most frequently mutated gene after TP53 (20). The Notch signaling pathway is also altered in HNSCC, with changes in gene copy number and expression of Notch pathway components such as JAG1 and JAG2 ligands. These changes activate the Notch signaling pathway, as demonstrated by the activation of HES1/HEY1 genes, which are effectors of the Notch pathway. Additionally, the role of Notch in HNSCC is regulated by the p53-related transcription factor p63, which acts as a suppressor of the *Notch1* gene (19). Therefore, in HNSCC, while a small percentage of Notch signaling pathway undergoes inactivating mutations via the Notch1 receptor, the majority exhibit changes in the expression and copy number of Notch1 signaling pathway receptors, ligands, and related effector genes. Although there is no direct literature reporting the interaction mechanism between Notch signaling and the cGAS-STING signaling. However, existing studies have found that the Notch pathway interacts with other signaling pathways, such as NFκB, to precisely regulate cell fate (19). The cellular-mesenchymal epithelial transition factor (c-MET) belongs to the receptor tyrosine kinase family, with hepatocyte growth factor (HGF) serving as its ligand. The c-MET/HGF/EGFR signaling pathway is frequently activated during tumorigenesis, promoting tumor formation, invasive growth, and metastasis. In most HNSCC cases, c-MET expression is upregulated (21). Upon specific binding to HGF in the extracellular domain, c-MET undergoes a conformational change and forms dimers that act on downstream effectors, including phospholipase Cγ (PLCγ), cytosolic Src kinase (C-Src), phosphoinositol-3-hydroxy kinase (PI3K), α-serine/threonine protein kinase (Akt), and mitogen-activated protein kinase (MAPK) pathways (22). Furthermore, c-MET establishes a crosstalk pathway with the epidermal growth factor receptor (EGFR) that promotes chemoresistance in HNSCC through the activation of downstream signaling molecules, including RAS, RAF, MEK1, MEK2, and ERK. The cMET/HGF/EGFR signaling pathway and the cGAS-STING signaling pathway are two important cellular signaling pathways that play crucial roles in regulating cell proliferation, immune responses, and inflammation. Although there is currently no literature directly addressing the interaction between c-MET/HGF/EGFR and the cGAS-STING signaling pathway, studies have found that MET-induced CD73 can inhibit the immunogenicity of STING-mediated EGFR mutant cancer (23), suggesting that the cMET/HGF/EGFR signaling pathway indirectly affects the cGAS-STING signaling pathway.

Given the reported associations of the TP53/Notch and c-MET/HGF/EGFR signaling pathways target the cGAS-STING signaling pathway in HNSCC, suggesting a pivotal role for the cGAS-STING pathway in the response of HNSCC. Previous studies have found that STING enhances cell death in HNSCC by regulating reactive oxygen species and DNA damage (24). Therefore, further investigation into its mechanism of action may provide new insights for the treatment of HNSCC.

## 3 The critical role of cGAS-STING signaling in HNSCC

The cGAS-STING signaling pathway plays a crucial role in the pathogenesis of HPV-positive HNSCC. During the immune response to viral infection, the DNA receptor cGAS recognizes viral DNA as a danger signal and, activates STING, which initiates downstream signaling, promoting the expression of type I interferons. Upon binding to their receptors on the cell membrane, interferons activate the Janus Kinase/Signal Transducer and Activator of Transcription (JAK/STAT) pathway, inducing the expression of ISGs, including cytokines and chemokines. Some of these ISGs directly contribute to the elimination of viruses, thereby triggering an immune response that mediates antiviral reactions (15). HPV, primarily HPV16, integrates into the host cell genome, leading to the expression of viral oncogenic proteins E6 and E7. HPV16 oncogene E7 can inactivate the cGAS-STING signaling pathway, impairing the immune response to viral infection (25, 26).

**Figure 3:**
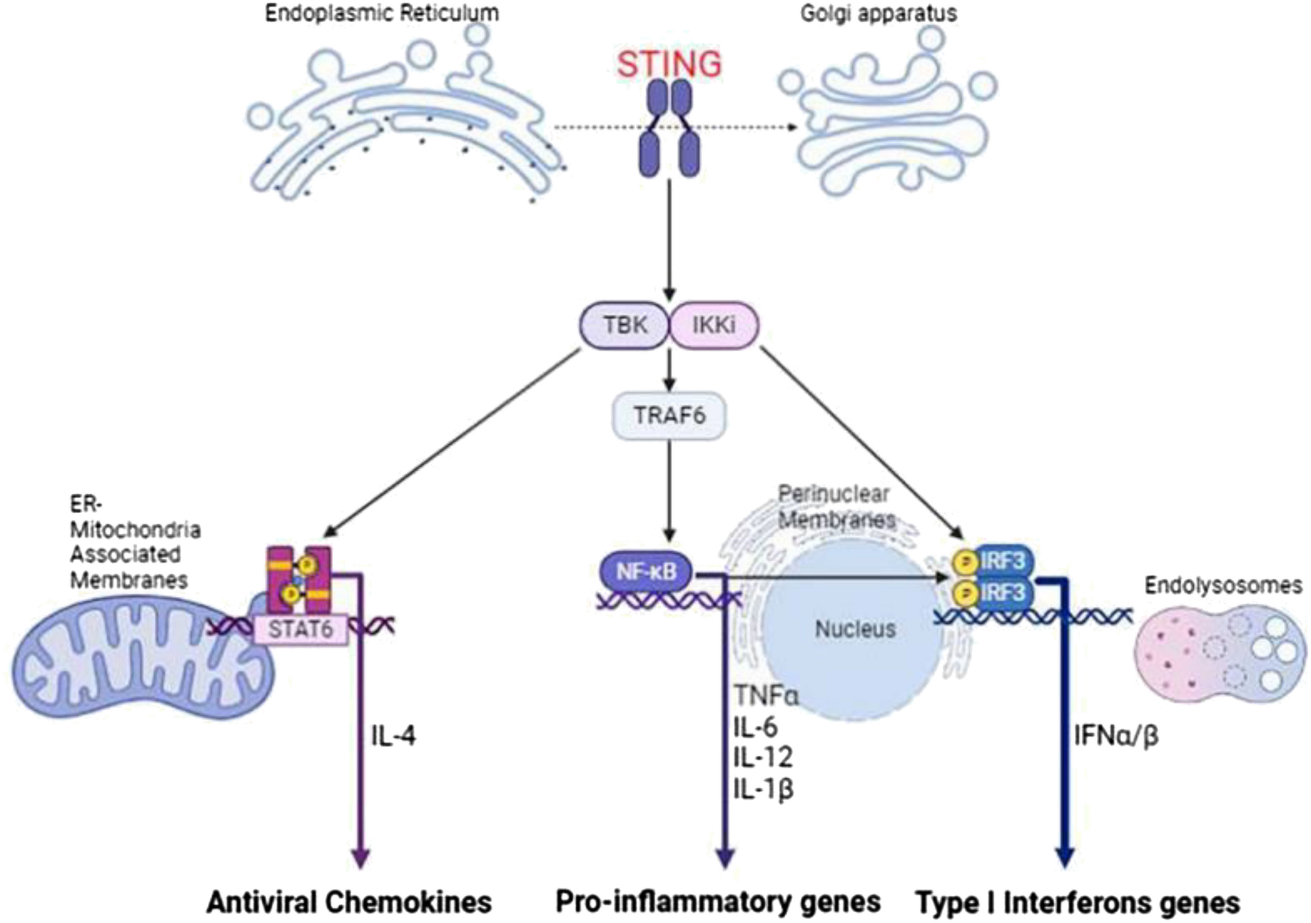
The downstream signaling pathways activated by STING. Upon activation and subsequent dimerization, STING translocates from the endoplasmic reticulum to the Golgi apparatus, where it recruits TBK1 and IKK, leading to the induction of a pro-inflammatory response through NF-κB activation, an IRF3-dependent type I interferon response, and upregulation of transcription of the antiviral transcription factor STAT6. This drawing was created using BioRender (https://biorender.com/).

Research has revealed STING is differentially expressed in HPV-positive and -negative HNSCC cell lines, they exhibit a gross functional defect in signaling through this pathway. Activation of STING in immune cell populations triggers antitumor mechanisms, thereby increasing the survival rate of HNSCC (27). In HPV-positive HNSCC, the activation of the cGAS-STING pathway is a critical mechanism for detecting and eliminating viral pathogens. Upon activation, STING coordinates the induction of type I interferons and other antiviral factors, promoting an immune response aimed at clearing viral infections. Additionally, STING participates in the induction of antiviral chemokines through the Signal Transducer and Activator of Transcription 6 (STAT6) and Interleukin-4 (IL-4) signaling pathways. Activation of this pathway leads to the production of chemokines that recruit immune cells to the site of infection, enhancing the antiviral immune response. A study by Li et al. (2018) (28) demonstrated that STING activation in macrophages induces the production of chemokines such as CCL17 and CCL22 through the STAT6 and IL-4 signaling pathways. The transcription factor NFκB can also be activated by STING, entering the nucleus and inducing the expression of pro-inflammatory cytokines such as Tumor Necrosis Factor Alpha (TNFα), IL-6, IL-12, and IL-1β. The most secreted cytokine in the STING pathway, IFN-β, can directly kill tumor cells and promote dendritic cell maturation for antigen presentation, triggering adaptive immune responses (**Figure 3**).

Recent studies have shown that the STING pathway is also effective in HPV-negative HNSCC models, suggesting that STING agonists may improve outcomes in both HPV-positive and HPV-negative HNSCC. Research using mouse models (24) indicates that STING activation enhances cell death through the regulation of reactive oxygen species and DNA damage. Moreover, studies (29, 30) have demonstrated that STING activation remains effective even in the absence of STING expression in cancer cells, highlighting the potential therapeutic benefit of STING agonists across different HNSCC types. Further details on the role of the cGAS-STING pathway in HPV-positive and HPV-negative diseases, and the potential of STING agonists in improving treatment outcomes, are warranted.

## 4 Therapy of HNSCC targeting cGAS-STING signaling

### 4.1 Clinical therapy of HNSCC

Currently, there is no effective specific screening strategy identified for HNSCC. While a small proportion of patients with oral precancerous lesions present with oral erythema or leukoplakia, which may be caused by cellular anisotropy, most patients are diagnosed at advanced stages. As a result, clinical data on precancerous lesions are still lacking. The current treatment for HNSCC is surgical excision or combination with adjuvant platinum-based chemotherapy and radiotherapy (CRT). The EGFR monoclonal antibody cetuximab is approved by the Food and Drug Administration (FDA) as a radiosensitizer, either alone or in combination with chemotherapy, for the treatment of patients with recurrent or metastatic disease (31). However, subsequent studies have found that its efficacy is not as good as that of cisplatin as a radiosensitizer (32, 33) (**Table 1**).

**Table 1:** Overview of clinical therapy of HNSCC.

Clinical Trial	Patient Characteristics	Cancer Site	Treatment Method
Phase II trial (2021) (34)	Advanced HNSCC patients	Head and neck	Weekly cisplatin chemotherapy (IMRT+C)
Phase III trial (published 2021) (35)	Advanced HNSCC patients	Head and neck	Gemcitabine plus Cisplatin (GP) and Fluorouracil plus Cisplatin (FP) chemotherapy regimens
Phase III trial (published 2021) (36)	Locally advanced or metastatic HNSCC	Head and neck	Capecitabine after radical radiotherapy
Phase III trial (RCT, 2021) (31)	Locally recurrent HNSCC	Head and neck	Endoscopic surgery vs. IMRT recurrence radiotherapy
Cetuximab vs. Cisplatin (32)	Recurrent or metastatic HNSCC patients	Head and neck	Cetuximab compared with cisplatin as radiosensitizer
Cetuximab vs. Cisplatin (33)	Advanced HNSCC patients	Head and neck	Comparison of efficacy of cetuximab and cisplatin

A recent phase II clinical trial published in the Journal of Clinical Oncology in 2021 found that subjects receiving weekly cisplatin chemotherapy (IMRT+C) maintained a good prognosis and substantial improvement in quality of life compared to patients treated with radiotherapy alone (34). The IMRT+C group met the expected endpoints, and a phase III study is expected to be conducted (**Table 1**).

For patients who are not suitable for radiotherapy alone, a phase III clinical trial published in the same journal reported on the efficacy of Gemcitabine plus Cisplatin (GP) and Fluorouracil plus Cisplatin (FP) chemotherapy regimens for patients with advanced HNSCC, enrolling 362 patients and showing that the GP regimen was beneficial, indicating that the GP regimen should be considered as a first-line treatment for palliative systemic chemotherapy for HNSCC (35). In contrast, a multicenter, randomized controlled, phase III clinical trial for refractory recurrent HNSCC or metastatic HNSCC showed that patients with locally advanced HNSCC who received capecitabine beat chemotherapy after completing radical radiotherapy further improved survival with less toxicity (36) (**Table 1**).

**Figure 4:**
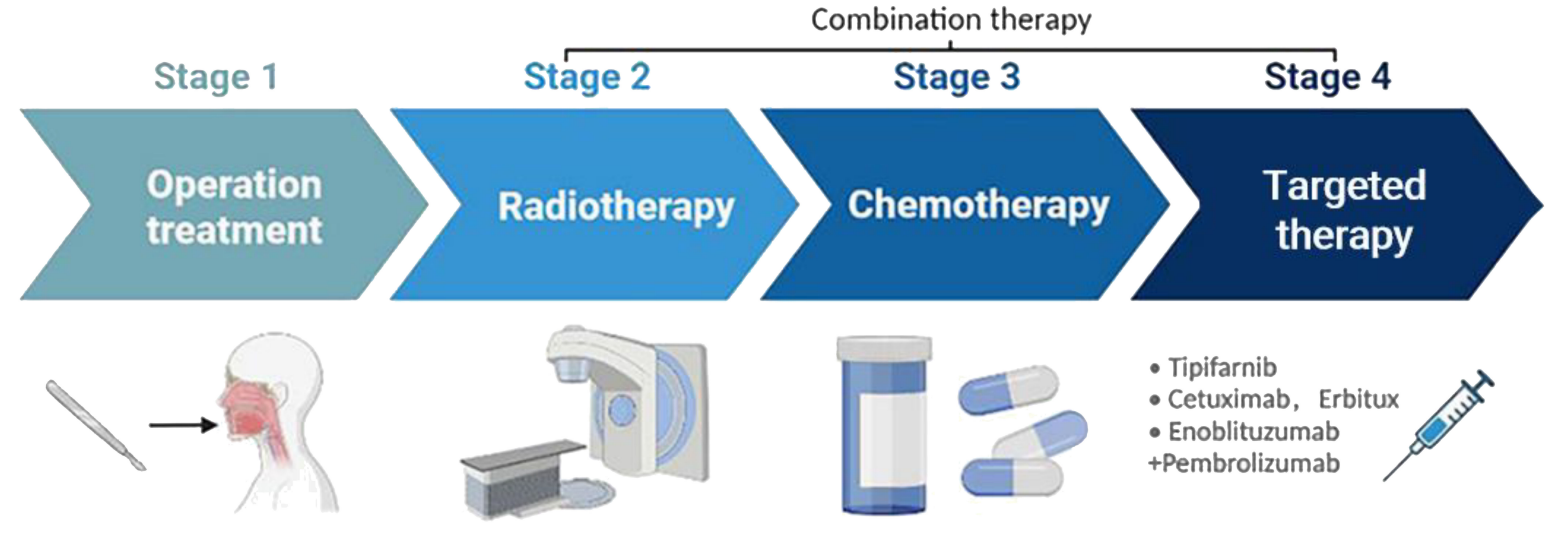
Treatment strategies for HNSCC. HNSCC is mainly treated through surgical resection, with adjuvant platinum-based CRT used alone or in combination. Immune checkpoint inhibitors such as tipifarnib, cetuximab, and endoblituzumab+pembrolizumab have been recently approved by the FDA for targeted treatment of HNSCC. This drawing was created using BioRender (https://biorender.com/).

Cisplatin-based radiochemotherapy is the standard of care for HNSCCs. Genotoxic therapies are potent inducers of the cGAS-STING signaling pathway and the antitumor IFN-1 response by generating cytosolic DNA fragments, either free or as micronuclei (37). The activation of this response by irradiation has also been demonstrated *in vitro* for HNSCC cells (38). This is relevant for achieving synergistic effects of radiotherapy and immune checkpoint inhibitors in HNSCC, of which the latter has been less effective to date. CRT can effectively reduce tumor volume, but it often leads to a deteriorated quality of life due to its low specificity and significant toxicities. A randomized controlled, phase III clinical study (39) aimed at exploring the efficacy of endoscopic surgery or IMRT recourse radiotherapy showed that surgery was superior to recourse radiotherapy for locally recurrent HNSCC. In terms of targeted immunotherapy, the CAPTAIN study (40) with carrilizumab and the POLARIS-02 study (41) with teraplizumab were the first to demonstrate the efficacy and safety of first-line use of anti-PD-1 monoclonal antibodies alone in the treatment of advanced HNSCC. Investigations have shown that HPV-positive patients have a better prognosis compared to the HPV-negative group, but currently, therapies fail in 15% of HPV-positive HNSCC with local recurrence or distant metastases (42). Since cetuximab was found to lead to worse overall and tumor-free survival rates in HPV-positive HNSCC compared to cisplatin, there is still no suitable treatment for all patients (32, 33). Therefore, there is an urgent requirement to develop new therapies for HNSCC (**Figure 4**).

### 4.2 Current therapy targeting cGAS-STING signaling

Studies have shown that activation of the cGAS-STING pathway can induce type I interferon production, leading to CD8+ T cell activation and tumor regression in mouse dendritic cells (43, 44). Woo et al (45) demonstrated that both STING and IRF3 double knockout mice lost the ability to produce interferon in response to tumors, indicating that the cGAS-STING pathway can be activated to treat tumors. Analysis of tumor specimens from HNSCC patients reveals that low STING expression is associated with worse outcomes (24). Saturated fatty acids induce the expression of Nucleotide-oligomerization domain (NOD)-like receptor subfamily C3 (NLRC3), thereby suppressing the STING-IFN-I pathway and reducing the immunogenicity of HNSCC (46). HPV-positive HNSCCs exhibit enhanced STING expression correlating with improved patient survival and potential benefits from STING agonist therapies in combination with standard treatments (27).

Several STING agonists, including 5, 6-dimethylxanthenone-4-acetic acid (DMXAA) and 10-carboxymethyl-9-acridanone (CMA), have been shown to possess anti-tumor activity and anti-pathogenic microbial properties (47). DMXAA, a flavonoid that was originally used as an anti-angiogenic agent (48), was found to be a direct ligand of STING in mice and induced phosphorylation of TBK1 and IRF3 to produce type I interferon (49). However, DMXAA acts as only a partial agonist in humans. This partial agonism is due to differences in the STING receptor between species; DMXAA effectively activates STING in mice but fails to fully activate human STING, which may explain its limited anti-tumor effects observed in human clinical trials. Despite this, DMXAA reached phase III in lung cancer clinical trials, though its mechanism of action against human lung cancer remains unclear (50). Targeted delivery of STING agonists to tumors can improve cancer immunotherapy (51). cGAMP, a natural ligand of STING, induces activation of the cGAS-STING pathway and has been found to have therapeutic effects in treating tumors by Deng et al (49, 52). The combined addition of 2’5’-cGAMP also enhanced the anti-tumor response, reduced tumor size, and increased the survival rate in mice. Moreover, the synergistic effects of cGAMP with 5-FU can reduce the therapeutic toxicity of 5-FU and combat tumors more effectively (53).

In addition, the expression of cGAS and STING proteins varies across different types of tumor cells. In human lung adenocarcinoma, the loss of cGAS has been shown to enhance tumor growth (54), while high expression of STING is associated with poor prognosis in colorectal cancer patient subgroups (55). These variations in expression highlight the potential of the cGAS-STING pathway as an attractive target for cancer therapy.

The presence of HPV is closely associated with patient survival and prognosis, possibly due to the different biological characteristics, such as invasiveness and immune response, induced by HPV infection. Investigations indicate tumor regression in HNSCC animal models receiving various STING agonists. HPV-negative HNSCC cells respond to cGAS-STING pathway activators, whereas HPV-positive HNSCC cells exhibit a poorer response. Additionally, STING activation enhances cetuximab-mediated NK cell activation and DC maturation, also correlated with HPV presence (29). cGAS-STING responses are dampened in high-risk HPV type 16 positive HNSCC (26). HPV16 drives cancer immune evasion through NLRX1-mediated STING degradation (56). The roles of HPV16 E6 and E7 in suppressing HNSCC responses are well-established. The presence of the HPV16 E7 oncogene inhibits cGAS-STING pathway activation (25), primarily due to the highly conserved LXCXE motif of the E7 oncogene obstructing cGAS-STING pathway activation (26). Furthermore, HPV E5 has been found to directly interact with STING, inhibiting downstream IFN signal transduction. By limiting the presentation of antigens on cell surfaces, HPV E5 may contribute to immune evasion (57). Therefore, targeting the HPV E7 oncogene and combining it with the use of STING agonists may represent a better therapeutic and preventative strategy for HNSCC.

Chromosomal instability is one of the key factors in cancer metastasis. HPV-negative and HPV-positive HNSCC cell lines exhibit similar numerical but distinct chromosomal aberrations, with higher genomic instability observed on chromosome 3 in HPV-positive cell lines compared to HPV-negative ones (58). Studies have found that chromosomal instability leads to the release of cytoplasmic DNA, activating the cytoplasmic DNA sensing pathway and promoting the metastatic ability of cancer cells. Specifically, chromosomal instability leads to DNA breaks and damage, resulting in the release of cytoplasmic DNA. The released DNA is recognized and activates the cGAS-STING signaling pathway in the cytoplasm, thereby triggering inflammatory responses and the activation of the transcription factor Signal Transducer and Activator of Transcription 3 (STAT3). Activation of STAT3 further promotes the expression of genes associated with metastasis, enhancing the metastatic ability of cancer cells. Furthermore, studies have also found that inhibiting the cytoplasmic DNA sensing pathway or repairing chromosomal instability can effectively suppress the metastatic ability of cancer cells (59, 60).

Based on the above, the cGAS-STING pathway may serve as an effective strategy for treating HNSCC. However, as illustrated by the examples above, the activity of the STING signaling in head and neck cancers varies due to different pathogenic factors. Therefore, personalized therapeutic strategy should be taken into account due to the different response to cGAS-STING agonists of HPV subtypes and tumor microenvironment. In addition, constructing corresponding disease models based on these factors is essential for exploring and elucidating the pathogenesis of HNSCC and developing personalized treatment strategies.

Traditional preclinical models include cell lines and murine models, which are used to mimic the molecular and cellular complexity of HNSCC. Konrad Klinghammer et al. discuss the existing cell lines, primary tumor cultures, and animal models, which are important for understanding HNSCC genetic variants and treatment resistance (61). Antonio Rueda-Domínguez et al. emphasize the efficiency of these preclinical models and focus future prospects on establishing new models capable of achieving metastasis in genetically modified mice (11). Additionally, personalized medicine is gaining traction in treating HNSCC, with advances in next-generation sequencing and multi-omic analysis improving personalized treatment development. Studies suggest that mimicking the natural tumor microenvironment through 3D systems and improved *in vivo* models can effectively enhance the efficacy of personalized medicine (62). However, further research and optimization are needed to address the distinct biological characteristics of HPV-positive and HPV-negative cases.

## 5 Discussion

HNSCC stands as a formidable oncological challenge with escalating incidence and mortality rates, posing a global public health concern. While smoking and alcohol abuse have traditionally been regarded as primary risk factors for HNSCC, the role of HPV infection in its pathogenesis cannot be understated. Distinct clinical characteristics and treatment responses between HPV-positive and HPV-negative HNSCC patients underscore the urgent need for personalized therapeutic strategies. Nevertheless, the diagnostic and therapeutic differentiation between HPV-positive and HPV-negative subtypes, as well as issues regarding drug resistance and recurrence, remain incompletely addressed.

Therapeutic targeting of the cGAS-STING signaling pathway represents a focal point in current HNSCC research. This pathway, integral to immune responses, plays a pivotal role in the development of HPV-positive HNSCC. Activation of cGAS-STING signaling pathway can induce apoptosis in tumor cells and promote activation of immune cells, thereby suppressing tumor growth and metastasis. However, current research predominantly focuses on animal models, necessitating further validation of its safety and efficacy in clinical applications.

Notably, chromosomal instability emerges as a key factor in HNSCC progression. Studies have revealed that chromosomal instability leads to the release of cytoplasmic DNA, activating the cGAS-STING signaling pathway and enhancing tumor cell metastatic capability, indicating that chromosomal instability (CIN) is also a potent regulator of cGAS-STING signaling in cancer cells. Activation of cGAS–STING-dependent inflammatory signaling downstream of CIN can exert both anti-tumoral and pro-tumoral effects in a cell-intrinsic manner. Expression of type I IFNs and activation of IFNAR/STAT1 induces the expression of multiple effectors, including pro-apoptotic and anti-proliferative genes, that are detrimental to tumor cell survival. Conversely, cGAS–STING-dependent activation of NC-NF-κB signaling can drive IL6/STAT3 signaling and EMT programs, which promote tumor growth and metastasis, respectively (60). Therefore, the application of therapeutic strategy targeting cGAS-STING should be curiously evaluated due to the contradictory effects, indicating that individualized therapy is of great importance.

In summary, while the cGAS-STING signaling pathway demonstrates immense potential in HNSCC therapy, further research and clinical practice are required to ascertain its efficacy and safety in both HPV-positive and HPV-negative patients. Moreover, comprehensive considerations encompassing personalized treatment, drug resistance, and metastasis are imperative for achieving improved clinical outcomes in HNSCC management.
